# Early intervention with extracellular vesicles derived from human umbilical cord mesenchymal stem cells enhances survival and functional recovery after spinal cord injury in rats

**DOI:** 10.3389/fphar.2025.1695914

**Published:** 2025-11-12

**Authors:** Barbara Porto Cipriano, Rachel Santana Cunha, Thaís Alves de Santana, Kátia Nunes da Silva, Erick Correia Loiola, Júlio César Queiroz Figueiredo, Elisama Araújo da Silva, Adne Vitória Rocha de Lima, Erik Aranha Rossi, Igor Campos da Silva, Ravena Pereira do Nascimento, Silvia Lima Costa, Zaquer Suzana Munhoz Costa-Ferro, Bruno Solano de Freitas Souza

**Affiliations:** 1 Center for Biotechnology and Cell Therapy, São Rafael Hospital, D’Or Institute for Research and Education (IDOR), Salvador, Brazil; 2 Gonçalo Moniz Institute, Oswaldo Cruz Foundation (FIOCRUZ), Salvador, Brazil; 3 Pioneer Science Initiative, D’Or Institute for Research and Education (IDOR), Rio de Janeiro, Brazil; 4 Laboratory of Neurochemistry and Cell Biology, Department of Biochemistry and Biophysics, Institute of Health Sciences, Federal University of Bahia, Salvador, Brazil

**Keywords:** spinal cord injury, mesenchymal stem cells, extracellular vesicles, survival, neuroinflammation

## Abstract

**Introduction:**

Spinal cord injury (SCI) is a devastating condition with high mortality and limited treatment options. Mesenchymal stem cell (MSC)-derived extracellular vesicles (EVs) have emerged as promising cell-free therapies due to their immunomodulatory and neuroprotective properties. Here, we evaluated the therapeutic potential of EVs derived from human umbilical cord MSCs in a rat model of SCI.

**Methods:**

Adult male Wistar rats were randomized into three groups: control, SCI, and SCI treated with a single intralesional dose of EVs. Locomotor recovery was assessed by the Basso, Beattie & Bresnahan (BBB) score, while survival, neuroinflammation, histological alterations, and biodistribution were systematically analyzed.

**Results:**

EV administration improved 30-day survival, and locomotor performance from day 7, and was associated with sustained reductions in pro-inflammatory cytokines (IL-1β, TNF-α) alongside increased levels of anti-inflammatory cytokines (IL-10) and neurotrophic factors (BDNF). Histological and immunofluorescence analyses showed attenuated microglial activation and astrocytic reactivity, accompanied by reduced lesion size and glial scar formation. *In vivo* imaging demonstrated accumulation of labeled EVs at the injury site, with peak retention at day 7 post-injection.

**Discussion:**

Together, these findings demonstrate that early intralesional delivery of MSC-EVs enhances survival, modulates the inflammatory response, and promotes functional recovery after SCI, supporting further translational development of EV-based interventions for SCI.

## Graphical Abstract

**Figure:**
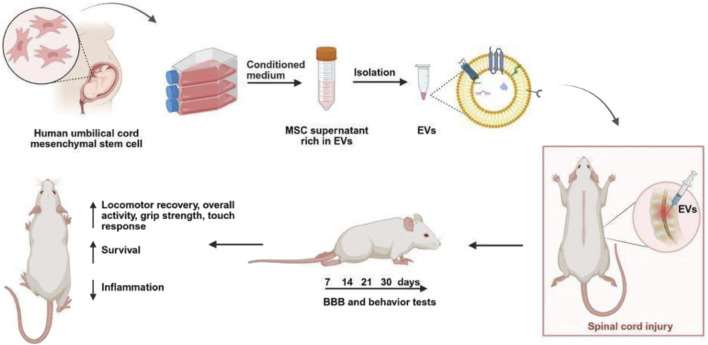

## 1 Introduction

Spinal cord injury (SCI) remains a major public health challenge, both in terms of mortality and morbidity, due to its high incidence and the scarcity of effective therapeutic options (Courtine and Sofroniew, 2019). Most cases arise from traumatic events leading to spinal fractures and/or dislocations, often resulting in permanent paralysis due to the limited regenerative capacity of the central nervous system (CNS) (Holmes, 2017). Mortality is particularly elevated in the first year after injury - largely due to respiratory complications, infections, and thromboembolic events - and survival rates of SCI patients remain reduced compared with the general population despite advances in acute care and rehabilitation, reinforcing the urgent need for interventions capable of mitigating early mortality while promoting functional recovery (Zadra et al., 2024).

Following the primary insult, SCI initiates a cascade of secondary injury mechanisms that amplify tissue damage and neurological deficits (Eli et al., 2021). Disruption of the blood-spinal cord barrier permits the infiltration of peripheral immune cells and plasma proteins, which synergize with the rapid activation of resident microglia (Sterner et al., 2023). Activated microglia, together with infiltrating macrophages, amplify tissue damage by releasing pro-inflammatory cytokines, ROS, and proteases. In parallel, astrocytes undergo reactive transformation, initially attempting to restore homeostasis but later contributing to glial scar formation and deposition of inhibitory extracellular matrix molecules (Tran et al., 2018). Ultimately, these events converge to neuronal death and glial cell activation, particularly of astrocytes, microglia, and endothelial cells, which in turn release pro-inflammatory cytokines and chemokines, amplifying a widespread inflammatory response (Zhang M., et al., 2021). Excitotoxicity, oxidative stress, ischemia, and edema further drive neuronal and oligodendrocyte death. Ultimately, SCI promotes a hostile inflammatory microenvironment that hampers regeneration, promotes gliosis, and creates long-lasting barriers to functional recovery (Zhang M. et al., 2021; Li et al., 2017).

Intercellular communication plays a central role in orchestrating these injury responses, with extracellular vesicles (EVs) emerging as key mediators of crosstalk between neurons, glia, endothelial cells, and infiltrating immune cells (Cao et al., 2025). According to the International Society for Extracellular Vesicles (ISEV), EVs comprise a heterogeneous population of membrane-bound vesicles (30–1000 nm in diameter) secreted by virtually all cell types. EVs carry proteins, lipids, and nucleic acids, including mRNAs and microRNAs, that can either propagate inflammation or promote repair, depending on their cellular origin (Welsh et al., 2024; Ragni et al., 2025; Sohrabi et al., 2022). Harnessing this natural signaling system, therapeutic strategies have increasingly focused on mesenchymal stem cell (MSC)-derived EVs, which combine the neuroprotective and immunomodulatory effects of MSCs with the practical advantages of a cell-free product (Volarevic et al., 2017; Wang et al., 2023).

Therapeutic efficacy has been previously demonstrated in MSC-based therapy studies using cells obtained from different sources, including bone marrow-derived MSCs (BM-MSCs) (Huang et al., 2023), adipose tissue-derived MSCs (ASCs) (Vialle et al., 2023), and human umbilical cord-derived MSCs (Sun et al., 2023). In SCI, most preclinical studies with MSC-EVs have primarily focused on motor recovery and histological outcomes. However, a translational gap remains: while SCI in patients is strongly associated with high early mortality due to systemic complications, most preclinical studies have limited their analyses to motor and histological endpoints. Whether EV-based interventions can also extend survival has not been addressed. Here, we evaluated the therapeutic efficacy of MSC-derived EVs in a rat model of acute SCI, focusing on survival, locomotor recovery, neuroinflammation, and neurotrophic signaling.

## 2 Materials and methods

### 2.1 MSC isolation and culture

Human Wharton’s jelly-derived MSCs were obtained and banked under approval of the Research Ethics Committee of São Rafael Hospital (CAAE: 09803819.30000.0048), with written informed consent provided by all participants. MSCs were sourced from the study biorepository at the Center for Biotechnology and Cell Therapy, Hospital São Rafael (D’OR Institute for Research and Education) at passage 3 (P3). Approximately 6 × 10^3^ cells/cm^2^ were seeded into T175 culture flasks and expanded in low-glucose DMEM (Gibco), supplemented with 2 mM L-glutamine (Gibco), Pen-Strep (Gibco), and 3% platelet lysate. Cultures were maintained at 37 °C in a humidified atmosphere with 5% CO_2_, and the medium was replaced every 3–4 days. At 70%–80% confluence, cultures were washed and maintained for 48 h in complete low-glucose DMEM without platelet lysate. The conditioned medium was then collected for EV isolation.

### 2.2 MSC characterization

#### 2.2.1 Immunophenotyping

MSCs were characterized by flow cytometry using a Navios cytometer (Beckman Coulter) and the BD Stemflow™ hMSC Analysis Kit. Cells were stained with antibodies for positive markers (CD73, CD90, CD105) and negative markers (CD34, CD45, CD11b, CD19, HLA-DR). After incubation and washing, samples (≥10,000 events) were analyzed with Kaluza software. MSCs were defined as CD73^+^CD90^+^CD105^+^ and negative for hematopoietic/endothelial markers. Appropriate controls were included for gating and compensation.

#### 2.2.2 Multi-lineage differentiation

MSCs were induced to differentiate into osteogenic, chondrogenic, and adipogenic lineages using commercial kits (StemPro, Thermo Fisher). Differentiation was confirmed by Alizarin Red staining (osteogenesis), Alcian Blue (chondrogenesis), and Oil Red O (adipogenesis), after 14 days (osteogenic and adipogenic) or 21 days (chondrogenic). Representative images were acquired with a Nikon Eclipse Ti1 inverted microscope (Nikon, Tokyo, Japan).

#### 2.2.3 Cytogenetic analysis

Cytogenetic evaluation was performed by G-band karyotyping to detect structural or numerical chromosomal alterations. MSCs were cultured at 4000 cells/cm^2^ and, at 80% confluence, treated with 0.1 μg/mL colcemid (Gibco). Cells were exposed to hypotonic 0.075 M KCl, fixed with Carnoy’s solution (3:1 methanol:acetic acid), and slides were aged at 60 °C for 16 h before GTG banding. Twenty metaphases were analyzed under a BX61 microscope (Olympus) with a digital imaging system (Applied Spectral Imaging, Carlsbad, CA), and images were processed with Lucia Karyo software (Lucia Cytogenetics, Prague, Czech Republic).

#### 2.2.4 Functional assays

The immunomodulatory potential of MSCs was evaluated by co-culture with activated lymphocytes. PBMCs (2 × 10^5^/well) were stimulated with CD3/CD28 beads (Thermo Fisher Scientific, 1:5 bead-to-cell ratio) and co-cultured with mitomycin C-treated MSCs at a 1:10 MSC:PBMC ratio, as previously described (Silva, et al., 2025). After 5 days at 37 °C/5% CO_2_, supernatants were harvested for cytokine quantification. TNF-α and IL-10 levels were measured by ELISA (R&D Systems, Minneapolis, MN, United States), with absorbance read at 450 nm (540 nm correction) on a GloMAX Explorer. Senescence was assessed with the CellEvent™ Senescence Green Detection Kit (Thermo Fisher) following manufacturer’s instructions. Nuclei were counterstained with Hoechst (1:1000). Hydrogen peroxide–treated MSCs (100–200 μmol/L for 2 h at 37 °C) served as positive controls. Quantification was performed in quadruplicate, analyzing 20 quadrants per well using an Operetta high-performance microscope (PerkinElmer, Waltham, MA, United States). Images were analyzed with Harmony 3.8 software (PerkinElmer), and data were expressed as mean ± SD of SA-βGal-positive cells.

### 2.3 EV isolation

The conditioned medium was centrifuged at 3000 *g* for 30 min at 4 °C to remove debris and filtered through a 0.22 μm membrane. Supernatants were concentrated using Amicon^®^ Ultra-15 Centrifugal Filter Units (Merck Millipore, Cork, Ireland), with 100 kDa pore, followed by overnight incubation with the Total Exosome Isolation reagent (Thermo Fisher Scientific) according to manufacturer’s instructions. The mixture was centrifuged at 10,000 × g for 1 h, and the resulting EV pellet was resuspended in PBS. EVs were stored at −80 °C until use.

#### 2.3.1 EV characterization

Nanoparticle tracking analysis (NTA) was performed with a ZetaView PMX-220 system (Particle Metrix). Measurements were taken in triplicate across 11 positions. Calibration was performed with 100 nm polystyrene beads, and data were processed with ZetaView software. Protein concentration was determined using the Pierce BCA Protein Assay Kit (Thermo Fisher Scientific). To evaluate morphology, transmission electron microscopy (TEM) was conducted using a JEOL 1230 microscope at 80 kV. Samples (10 µL) were adsorbed onto formvar carbon-coated copper grids for 5 min, stained with 2% uranyl citrate for 1 min, and air-dried for 24 h, as previously described (Costa-Ferro et al., 2024). The presence of EV markers (CD9, CD63, CD81) was confirmed with the ProcartaPlex™ Human Exosome 6-plex Panel on a Luminex MAGPIX system (Luminex Corporation, Austin, TX, United States). Data were analyzed using Luminex software.

### 2.4 SCI model

All animal protocols were approved by the Ethics Committee of Fiocruz-BA (#020/21, issued on March 15, 2021). Adult male Wistar rats (45–50 days, 212.6 ± 5.8 g) were maintained under standard conditions (22 °C ± 1 °C, 60% humidity, 12 h light/dark cycle) with free access to food and water. After 1 week of acclimatization, the rats were randomly assigned to experimental groups: Control (no injury), SCI (vehicle only), and SCI + EV (treated with a single intralesional dose of EVs, 1 × 10^9^ particles in 10 μL PBS).

Surgical procedure: SCI was induced as described previously (Vanický et al., 2001). Briefly, rats were anesthetized with ketamine (80 mg/kg) and xylazine (10 mg/kg, i. p.), and a midline incision was made to expose T9. After laminectomy, a groove was drilled in T11 to guide insertion of a 2-French Fogarty catheter (Baxter Healthcare Corporation, Irvine, CA) into the epidural space. The balloon was inflated with 10 μL saline for 90 s, then deflated and removed. EVs were administered at the lesion site. Then, the catheter was deflated and removed and the soft tissues and skin were sutured in anatomical layers. The SCI model was established and EVs were injected into the lesion site. Post-injury, the muscle and skin layers were sutured with 5–0 silk sutures, and the rats were placed on a heating pad to maintain body temperature during recovery.

Postoperative care: Animals received postoperative care including a single dose of dipyrone (125 mg/kg, i. p.) for analgesia, hydration, daily manual bladder expression until spontaneous voiding, and monitoring for signs of distress or complications. Animals were euthanized at 24 h, 7 days, and 30 days post-injury for tissue collection.

#### 2.4.1 *In vivo* biodistribution of DiR-labeled EVs

The biodistribution of MSC-derived EVs was assessed using the near-infrared lipophilic dye 1,1′-dioctadecyl-3,3,3′,3′-tetramethylindotricarbocyanine iodide (DiR; Invitrogen, United States), following labeling procedures previously described by our group (Costa-Ferro et al., 2024; Costa-Ferro et al., 2025). DiR-labeled EVs (1 × 10^9^ particles in 10 µL PBS) were administered intralesionally immediately after SCI. *In vivo* fluorescence imaging was performed at 1, 7, and 14 days post-injection using the AMI HTX System (Spectral Instruments Imaging, Tucson, AZ, United States) with excitation/emission filters of 710/770 nm, and the fluorescence signal was quantified as total radiant efficiency using AMIView software. Identical regions of interest (ROIs) were applied for all animals, and background signal from control animals was subtracted.

#### 2.4.2 Basso-beattie-bresnahan (BBB) scoring

The recovery of hind limb motor function on the 1st, 7th, and 14th days after injury was evaluated using the BBB scoring system (n = 7/group). During the test, each rat was placed in an open field and allowed to walk freely for a 5-min observation period. Hind limb movement was assessed on a 0–21 point scale. A score of 0 indicates complete paralysis with no observable leg movement, and a score of 21 represents normal locomotion. The BBB assesses various aspects of motor function, including hindlimb movement, joint movements, stepping ability, trunk stability, and coordination during open-field locomotion (Basso et al., 1995).

#### 2.4.3 Survival and behavioral analyses

Clinical and general health parameters were assessed daily according to a 0–3 clinical scoring system (n = 7/group), assessed by two observers. Neuromuscular and autonomic parameters, including touch response, tail grip, tremors, muscle tone, piloerection, hypothermia, diarrhea, cyanosis, changes in urine color, and general activity were assessed daily for 1, 7 and 14 days following the administration of EVs using analog scales (n = 7/group) (Gale et al., 1985). Animal survival and behavioral recovery were monitored for up to 30 days after SCI, by two observers. Survival data were analyzed using the Kaplan–Meier estimator, and differences between groups were determined using the log-rank (Mantel–Cox) test (n = 20/group).

### 2.5 Quantitative real-time polymerase chain reaction (qRT-PCR)

Initially, spinal cord tissues were collected and snap-frozen. Total RNA was extracted using TRIzol™ (Thermo Fisher Scientific, Waltham, MA, United States) following the manufacturer’s instructions. After extraction, the RNA purity was measured photometrically using NanoDrop™ 1000 (Thermo Fisher Scientific, Waltham, MA, United States). Then, RNA samples (1.5 μg per sample) were converted to cDNA using a high-capacity cDNA reverse transcription kit (Thermo Fisher Scientific, Waltham, MA, United States). Aiming to quantify mRNA expression, TaqMan Master Mix and TaqMan™ probes were used at a final volume of 10 μL, following the manufacturer’s instructions (all from Thermo Fisher Scientific, Waltham, MA, United States), for detection of interleukin 1 beta (IL-1β, Rn00580432_m1), tumor necrosis factor (TNF-α, Rn99999017_m1), interleukin 10 (IL-10, Rn01483988_g1) and brain-derived neurotrophic factor (BDNF, Rn02531967_s1) genes. Finally, the PCR amplification was performed in a ABI7500 real-time PCR system (Applied Biosystems, Foster City, CA, United States) under standard thermal cycling conditions. The thermal cycling was performed according to the manufacturer’s recommendations. Relative mRNA expression levels were calculated using the comparative ΔΔCt method. Briefly, the Ct values of target genes were normalized to GAPDH (ΔCt), and fold changes in expression were determined relative to the control group (ΔΔCt).

### 2.6 Histological analysis

Spinal segments around the lesion center were removed and fixed 48 h in 10% formaldehyde. After stepwise dehydration with different concentrations of ethanol solution, the samples were paraffin-embedded, continuous transverse sections were prepared, hematoxylin and eosin (H&E) staining was performed according to the manufacturer’s instructions and cut into 5 μm sections. Images were collected using a digital pathological section scanning system (Zeiss image.Z.2, Oberkochen, Germany). The cellular infiltrates and necrosis were classified as mild (0), moderate (1), and extensive (2). The prepared slides were then examined under a light microscope. Cellular infiltration and tissue necrosis were semiquantitatively classified as mild (0), moderate (1), or extensive (2). All histological evaluations were performed in a blinded manner by an experienced pathologist.

### 2.7 Immunofluorescent staining

For the immunofluorescence staining, spinal cord segments, sectioned into paraffin sections measuring 5 μm in thickness, were deparaffinized using xylene (Exodo Cientifica) and rehydrated after successive washes using different concentrations of alcohol (100%, 90%, 70% and 30%) for later heat-induced antigen retrieval using citric acid for 25 min. After this time, spinal cord samples were incubated overnight with the primary antibody anti-GFAP (dilution of 1:400, Z0334, Invitrogen), anti-IBA1 (dilution of 1:200, ab178680, Abcam), and anti-CSPG (dilution of 1:100, SAB5700198, Merk). Following a thorough washing process, the sections were incubated with a secondary antibody (dilution of 1:800, A11011, Invitrogen) for 1 h at room temperature. Nuclei were stained with DAPI (Fluoroshield™ with DAPI, Sigma). Then, the presence of fluorescent cells was observed using a Confocal fluorescence microscope (Zeiss, Oberkochen, Germany), while the quantification of the number of fluorescent cells was performed using the CellInsight CX7 LED Pro HCS Platform (Thermo Fisher Scientific).

### 2.8 Statistical analyses

All statistical analyses were performed using GraphPad Prism 8 (GraphPad Software, United States). Data are expressed as mean ± SD from independent biological replicates, and statistical significance was set at p ≤ 0.05. Survival was analyzed by the Kaplan-Meier method and compared using the log-rank (Mantel–Cox) test. Behavioral and locomotor data, including BBB and clinical scoring, were analyzed by two-way repeated-measures ANOVA (treatment × time) followed by Dunnett’s *post hoc* tests. Gene expression (RT-qPCR) and EV quantifications were analyzed by one- or two-way ANOVA with Tukey or Dunnett *post hoc* tests. Comparisons between two groups were performed using unpaired t-tests. When data did not meet normality assumptions (Shapiro–Wilk test), non-parametric alternatives (Mann–Whitney or Kruskal–Wallis with Dunn’s multiple comparison) were applied, as indicated in the figure legends.

## 3 Results

### 3.1 Manufacturing and characterization of MSCs and EVs

**FIGURE 1:**
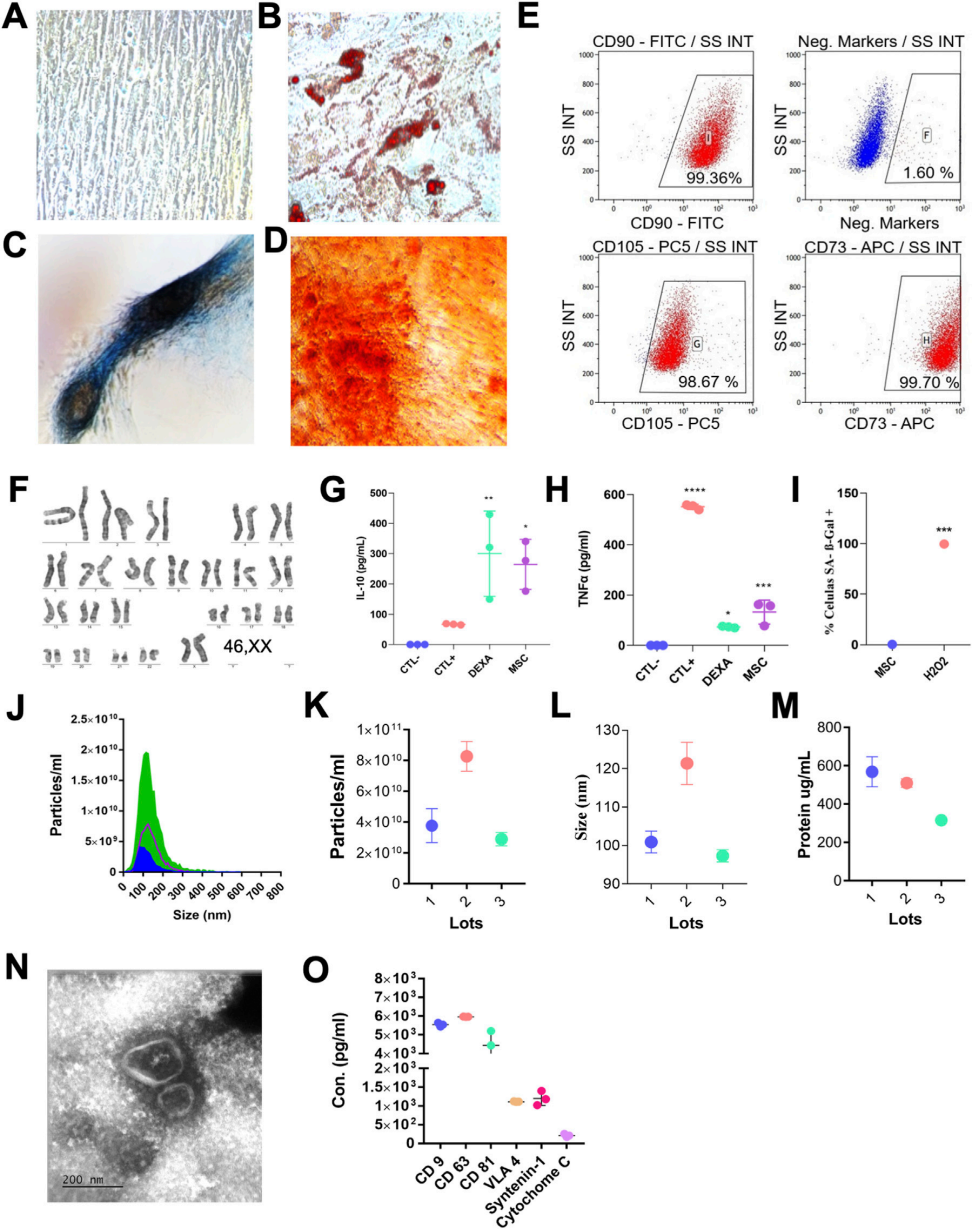
Characterization of MSCs and EVs. **(A)** Representative image of undifferentiated MSCs and differentiated MSCs toward adipogenic **(B)**, chondrogenic **(C)**, and osteogenic **(D)** lineages *in vitro*. **(E)** Representative flow-cytometry dot plots showing positive expression of MSC surface markers (CD73, CD90, CD105) and lack of hematopoietic markers (CD34, CD45, CD11b, CD19, HLA-DR). Data representative of three independent analyses. **(F)** Representative G-banded karyotype confirming genomic stability (46,XX). **(G,H)** Immunomodulatory potency of MSCs assessed in co-culture with activated PBMCs, showing IL-10 upregulation and TNF-α suppression. Experimental conditions: Ctl–, unstimulated PBMCs; Ctl+, PBMCs stimulated with anti-CD3/CD28; Dexa, PBMCs stimulated with anti-CD3/CD28 and treated with dexamethasone; MSC, PBMCs stimulated with anti-CD3/CD28 and co-cultured with MSCs for 5 days. Cytokines were quantified by ELISA. **(I)** Quantification of SA-β-gal-positive cells showing low senescence levels in MSCs compared with H_2_O_2_-treated controls. **(J–L)** Nanoparticle tracking analysis (NTA) showing EV particle size distribution and concentration obtained from three independent EV batches. **(M)** Total protein content of EV samples (µg/mL); values represent mean ± SD of three EV lots. **(N)** Representative transmission electron microscopy image showing typical cup-shaped EV morphology (50–150 nm; scale bar, 200 nm). **(O)** EV protein marker profiling showing strong expression of canonical exosomal markers (CD9, CD63, CD81) together with VLA-4 and syntenin-1, and minimal levels of cytochrome C (assessed by Luminex; n = 3 EV lots). Statistical analysis: **(G,H)** one-way ANOVA with Tukey multiple-comparison test versus Ctl+; **(I)** unpaired t-test (two-tailed). Data are presented as mean ± SD; n = 3 biological replicates per condition. *p < 0.05; **p < 0.01; ***p < 0.001.

MSCs demonstrated plastic adherence and exhibited the typical spindle-shaped morphology (Figure 1A). Their multipotency was confirmed by successful differentiation into osteogenic, adipogenic, and chondrogenic lineages (Figures 1B–D). As analyzed by flow cytometry, MSC surface negative markers (CD34, CD45, CD11b, CD19, HLA-DR) were minimally expressed and positive for MSC markers (CD73, CD90, CD105) were prominently expressed, confirming a characteristic MSC immunophenotype (Figure 1E). Cytogenetic analysis by G-banding revealed genomic stability, with a normal female karyotype (46,XX) and no structural or numerical abnormalities detected (Figure 1F).

To assess the biological activity of the MSCs, their immunomodulatory function was evaluated through co-culture assays with activated PBMCs. The cells showed the ability to inhibit TNF-α and induce IL-10 expression (Figures 1G,H), supporting their anti-inflammatory potential. Furthermore, senescence-associated β-galactosidase staining revealed low levels of cellular senescence (Figure 1I), indicating that the MSC population maintained proliferative capacity, genomic stability, and overall cellular fitness, consistent with a therapeutically competent phenotype suitable for EV production.

EVs were isolated from the supernatants of MSC cultures across three independent batches. The EV samples displayed consistent size profiles, with nanoparticle tracking analysis (NTA) indicating a size range between 35 and 200 nm (Figures 1J–L). Protein concentration of the EV preparations ranged from 316 ± 6 to 568.5 ± 55.5 μg/mL (Figure 1M). Transmission electron microscopy confirmed the typical cup-shaped morphology of EVs, revealing bilayer membrane structures with heterogeneous sizes (Figure 1N). Multiplex immunoassays demonstrated that EVs were enriched in canonical exosomal markers (CD9, CD63, and CD81, syntenin-1, and VLA-4, while the non-EV contaminant cytochrome C was either minimally detected or undetectable (Figure 1O).

### 3.2 EV therapy promotes functional recovery post-SCI model

**FIGURE 2:**
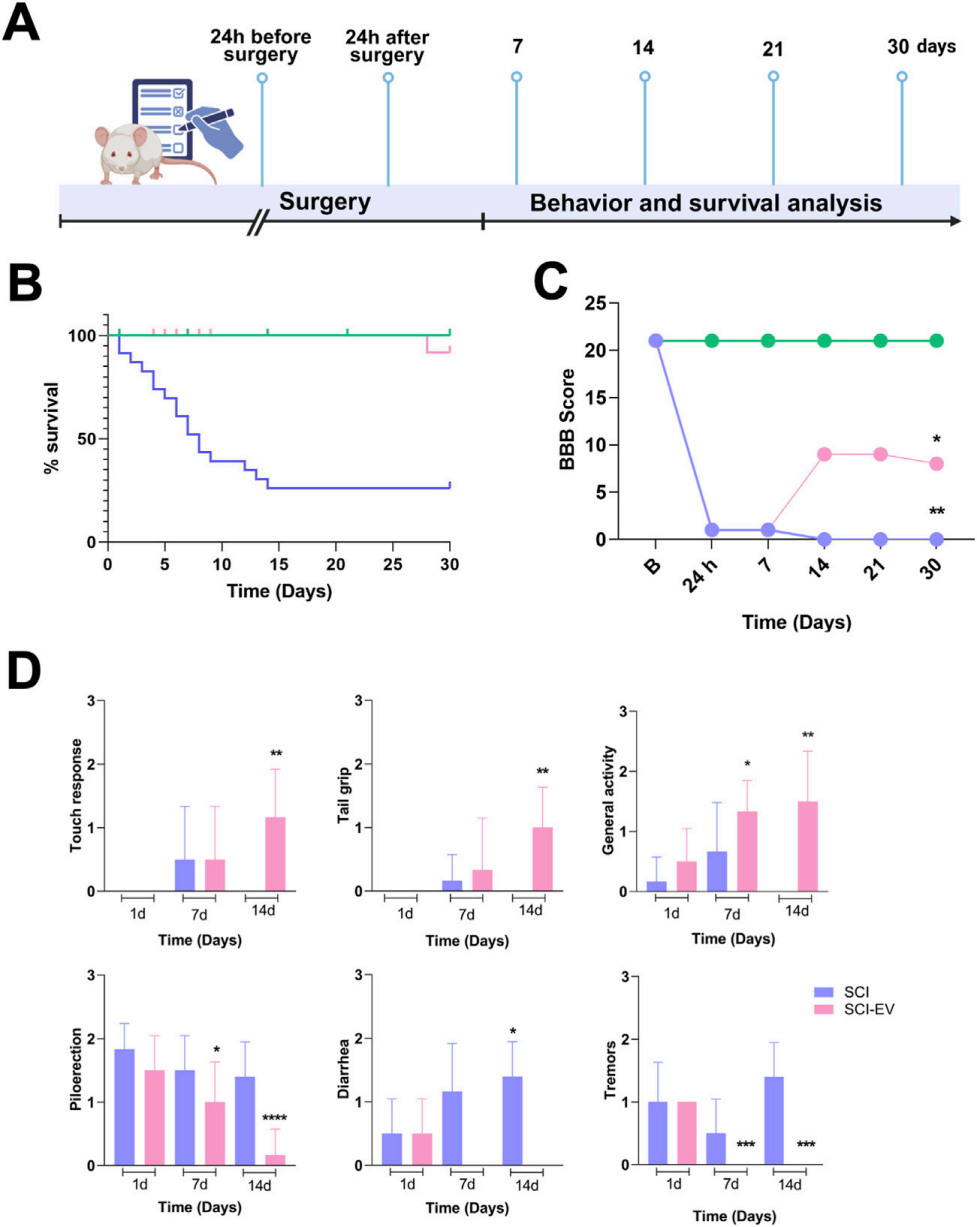
EVs improve survival and locomotor recovery after SCI. **(A)** Experimental timeline showing EV administration (1 × 10^9^ particles/rat, administered intralesionally, immediately post-injury) and follow-up up to 30 days. **(B)** Survival curves of control (green), SCI (blue), and SCI-EVs (pink) groups showing markedly reduced survival after SCI, which was partially improved by EV treatment. Statistical analysis was performed using the log-rank (Mantel–Cox) test (n = 20 per group). *p < 0.05, **p < 0.01 vs. SCI. **(C)** Locomotor function assessed using the BBB scale by two independent blinded evaluators. SCI-EVs animals (pink) showed progressive functional improvement from day 14 onward compared to the SCI group (blue), while controls (green) maintained normal scores (n = 7 per group). **(D)** Clinical and behavioral recovery scoring according to a modified 0–3 scale (n = 7 per group). Upper panels: neuromuscular parameters (touch response, tail grip, general activity) were scored as 0 = severe impairment, 1 = moderate, 2 = mild, 3 = normal. Lower panels: autonomic/toxicity parameters (piloerection, diarrhea, tremors) were scored as 0 = normal, 1 = mild, 2 = moderate, 3 = severe. **(C,D)** Data are presented as mean ± SD. Statistical analysis was performed using two-way ANOVA followed by Dunnett’s *post hoc* test. *p < 0.05, **p < 0.01, ***p < 0.001 compared with the SCI group.

*In vivo*, we evaluated the neuroprotective efficacy of EVs in an SCI model, following the experimental timeline demonstrated in Figure 2A. Following SCI, untreated animals exhibited severe urogenital complications, including marked urinary retention and, in some cases, hematuria post-injury, often accompanied by lethargic behavior. Despite antibiotic administration, the health status of untreated animals progressively deteriorated. EV treatment was associated with improved survival rates over the 30-day follow-up compared with untreated SCI animals (Figure 2B). To further assess the functional impact of EV therapy, locomotor recovery was evaluated using the BBB scoring system at predefined time points after injury. EV-treated animals showed a significant and progressive improvement in BBB scores, with differences from the SCI group already evident at day 7 and sustained throughout the 30-day follow-up (Figure 2C). Moreover, a time-dependent improvement in clinical parameters was observed, compared with untreated SCI animals. EV-treated rats showed faster recovery of behavior parameter analyzed, including touch response, tail grip strength, general activity, reduced tremors, and improved autonomic regulation (Figure 2D).

### 3.3 EVs sustainably reduce inflammation and enhance neurotrophic support after the SCI model

**FIGURE 3:**
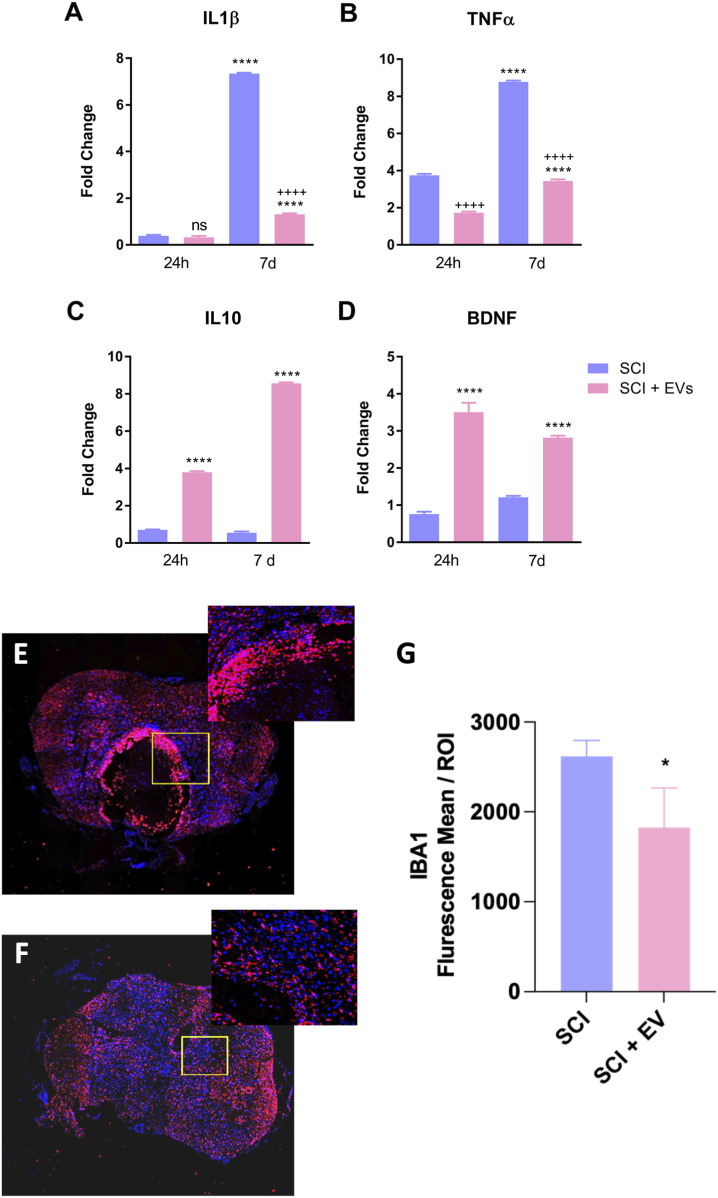
EV treatment modulates inflammatory cytokines and microglial activation after spinal cord injury. Relative gene expression of **(A)** IL-1β, **(B)** TNF-α, **(C)** IL-10, and **(D)** BDNF in SCI and SCI-EV groups was determined by RT-qPCR. Data were normalized to GAPDH and expressed as fold change relative to non-injured controls. **(E,F)**. Values represent mean ± SD (n = 7 per group/time point, one-way ANOVA with Tukey multiple-comparison test). Representative confocal images of IBA1 (red) and DAPI (blue) immunofluorescence in spinal cord sections from SCI **(E)** and SCI + EVs **(F)** groups at 7 days post-injury (200x magnification). Insets show higher magnification of the lesion border. **(G)** Quantification of IBA1 immunoreactivity (mean fluorescence intensity per ROI) showing decreased microglial activation in EV-treated rats (n = 5-7 per group, unpaired t-test, p < 0.05). ns = not significant; p < 0.05; p < 0.001 vs. SCI; “+” indicates downregulation relative to SCI; data presented as mean ± SD.

Figure 3 shows the effects of SCI-EVs treatment on inflammatory cytokines and the neurotrophic factor BDNF at 24 h and 7 days after SCI, expressed as fold change relative to the control group. At 24 h, IL-1β remained near baseline (ns), whereas TNF-α (p < 0.001) was already elevated in SCI. EV treatment markedly attenuated TNF-α (p < 0.001) expression while strongly increasing both IL-10 (p < 0.001) and BDNF (p < 0.001). At 7 days post-injury, these modulatory effects were maintained, with a pronounced reduction in IL-1β (p < 0.001) and TNF-α (p < 0.001) and a robust increase in IL-10 (p < 0.001) and BDNF (p < 0.001) compared to untreated SCI animals. Immunofluorescence analysis of IBA-1-positive microglia revealed a marked reduction in activated IBA-1 cells in the lesion area of animals treated with EVs (Figures 3E,F). Quantification at 7 days post-injury (Figure 3G) confirmed a significant decrease in IBA-1 immunoreactivity in the SCI-EVs group compared with SCI controls (p < 0.05), supporting the anti-inflammatory action of EVs in the injured spinal cord.

### 3.4 EVs suppress SCI-induced glial scar formation around the lesion site after SCI

**FIGURE 4:**
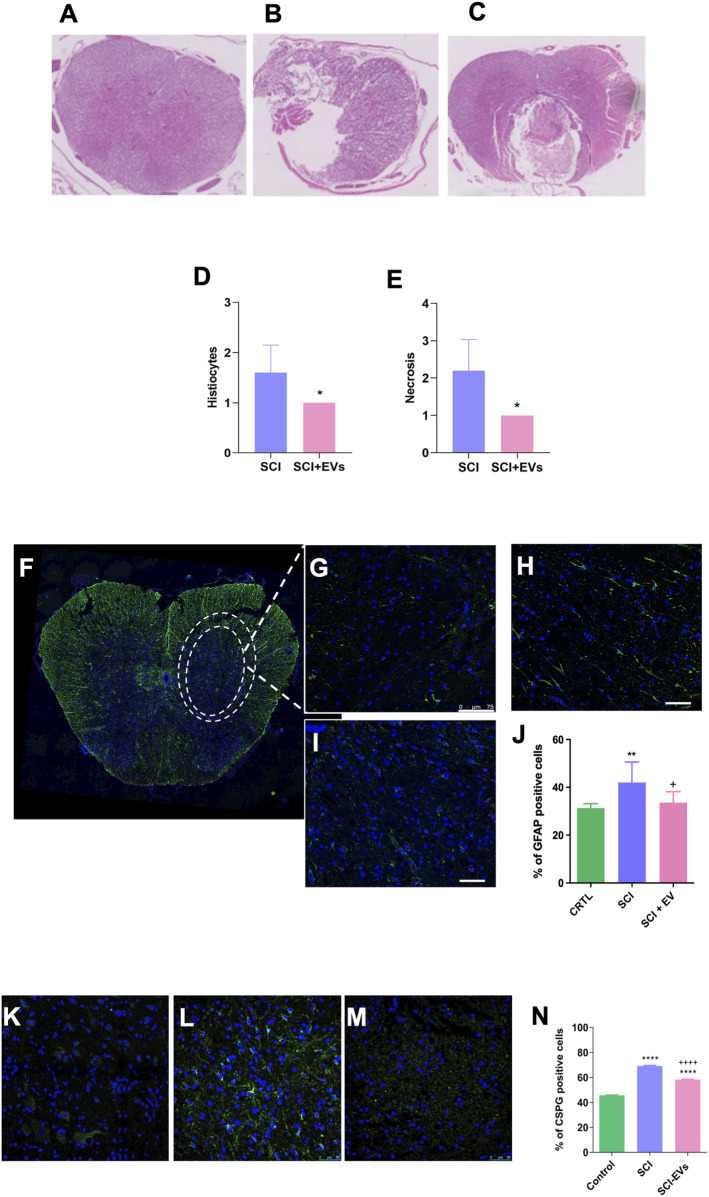
EV treatment reduces lesion severity, glial reactivity, and CSPG deposition after SCI. **(A–C)** Representative hematoxylin and eosin (H&E)-stained spinal cord sections from intact control, SCI, and SCI-EV groups at 7 days post-injury (200x magnification). Quantitative histological analysis reveals a significant reduction in histiocytic infiltration **(D)** and necrotic area **(E)** in the SCI-EVs group compared with SCI (p < 0.05). Representative immunofluorescence images of GFAP-positive astrocytes (green) with nuclear counterstaining (DAPI, blue) **(F,G)** intact control, **(H)** SCI, and **(I)** SCI-EVs (400x magnification). **(J)** Quantification of GFAP + astrocytes in the spinal cord. **(K–M)** CSPG representative immunofluorescence staining, with CSPG (green), with a nuclear counterstaining with DAPI (blue): **(K)** intact control, **(H)** SCI, and **(I)** SCI-EVs (400x magnification). **(N)** Quantification of CSPG-positive cells demonstrates a significant increase after SCI and partial attenuation following EV administration. Data are presented as mean ± SD (n = 5-7 per group). Statistical significance was defined as *p < 0.05; ***p < 0.001 and +++p < 0.001.“+” indicates downregulation relative to SCI.

Histological analysis at 7 days post-injury revealed extensive tissue damage and cavity formation in the SCI group, whereas animals treated with EVs showed a reduced lesion area and tissue preservation (Figures 4A–C). Semiquantitative assessment of histiocytic infiltration and necrosis in H&E-stained sections demonstrated a significant reduction in lesion severity in the SCI-EV group compared with untreated SCI animals (p < 0.05; Figures 4D,E). Immunofluorescence staining further demonstrated a marked accumulation of GFAP-positive astrocytes in the SCI group, consistent with glial scar formation (Figures 4F–I). In contrast, EV-treated animals (Figure 4I) exhibited attenuated astrocytic reactivity as reflected by the reduced density of GFAP-positive cells (***p < 0.001; Figure 4J). Additionally, immunolabeling for chondroitin sulfate proteoglycans (CSPGs, Figures 4K–M) revealed pronounced matrix deposition in the SCI group (Figure 4L), whereas EV administration substantially reduced CSPG accumulation (+++p < 0.001; Figure 4N). Quantitative analysis confirmed that CSPG expression was significantly higher in the SCI group compared with controls (****p < 0.0001), and attenuated following EV treatment, indicating mitigation of glial scar formation.

### 3.5 *In vivo* biodistribution of EVs after SCI

**FIGURE 5:**
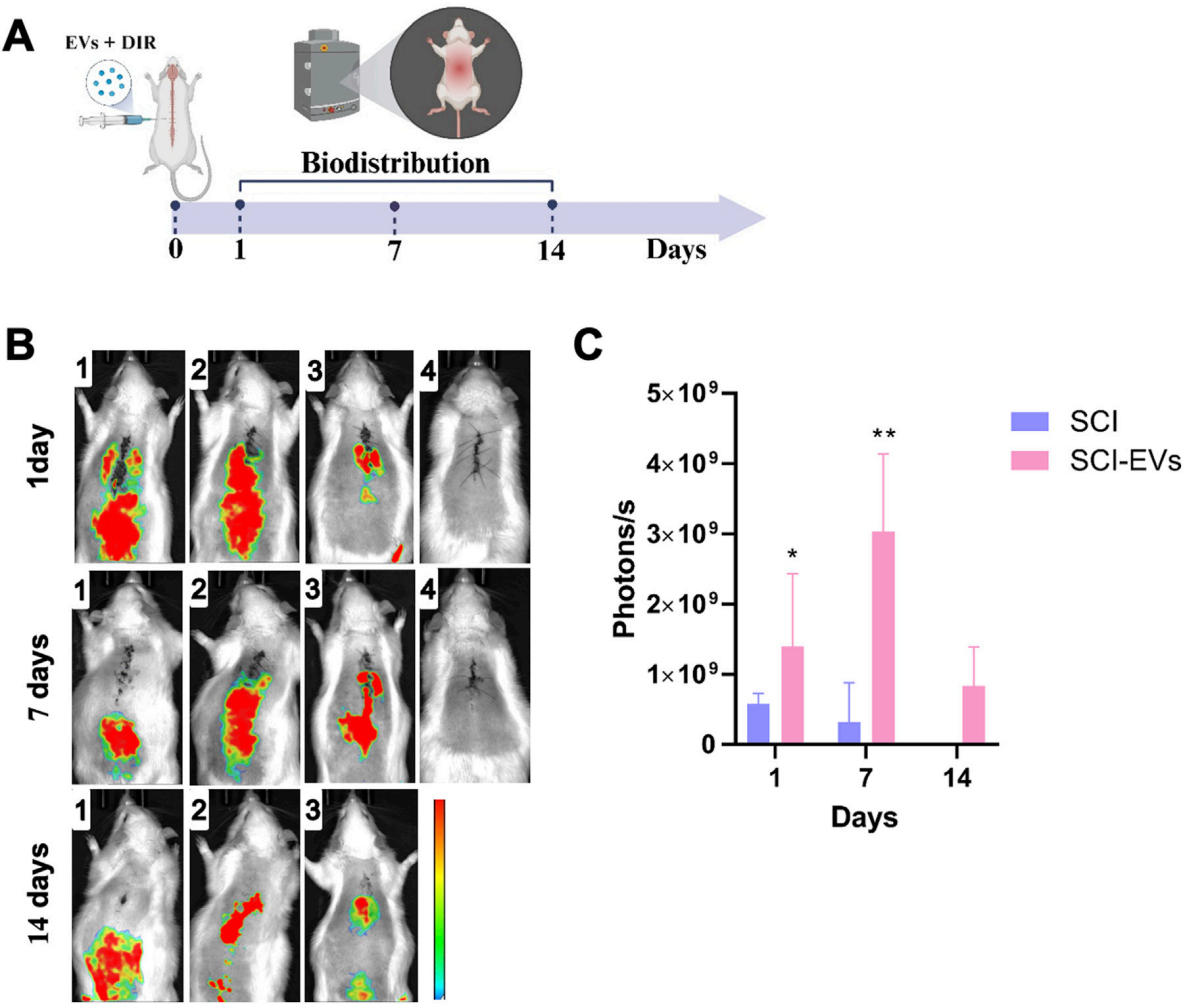
*In vivo* biodistribution of DiR-labeled EVs after local injection in SCI. **(A)** Schematic representation of the experimental timeline and marked imaging time points. EVs were labeled with the near-infrared dye DiR and locally injected at the lesion site in SCI rats. **(B)** Representative fluorescence images acquired at days 1, 7, and 14 post-injection. Each row represents the SCI-EVs group, and each column corresponds to a different animal (1–3) and control (4), showing fluorescence signals predominantly localized in the thoracic spinal cord region. **(C)** Quantification of photon emission revealed significantly higher fluorescence intensity in the SCI-EVs group compared to SCI. Data are presented as mean ± SD (SCI, n = 3; SCI-EVs, n = 6) and were analyzed using two-ANOVA followed by Tukey’s *post hoc* test. *p < 0.05; **p < 0.01.

*In vivo* tracking of DIR-labeled EVs demonstrated a selective accumulation at the spinal cord injury site in the SCI-EVs group compared with SCI controls (Figure 5). Representative fluorescence images (B) revealed a strong and localized signal in SCI-EVs-treated animals at days 1, 7, and 14 post-injection, with maximal intensity at day 7. Quantitative analysis (C) showed that, at day 1, photon emission was significantly higher in the SCI-EVs group (1.2 ± 0.6 × 10^9^ photons/s) compared with the SCI group (0.4 ± 0.1 × 10^9^ photons/s; p < 0.05). At day 7, the signal in the SCI-EVs group remained elevated (0.5 ± 0.2 × 10^9^ photons/s; p < 0.01). By day 14, the signal in the SCI-EVs group decreased (1.0 ± 0.5 × 10^9^ photons/s) but remained above baseline, whereas SCI controls presented minimal or undetectable fluorescence across all time points. These findings indicate a sustained retention of EVs at the lesion site, particularly during the first week after administration.

## 4 Discussion

SCI is a severe condition associated with high mortality, disability, and a substantial socioeconomic burden on patients’ families (Eli et al., 2021). The sudden onset and complex pathophysiology of SCI highlight the urgent need for improved therapeutic strategies. EVs from bone marrow, adipose, and umbilical cord MSCs have shown significant therapeutic potential (Mou et al., 2025). In this study, we demonstrate that early administration of MSC-EVs confers significant neuroprotection and functional benefits in a rat model of SCI. EV administration not only promoted partial motor recovery, as evidenced by improved BBB scores, but also mitigated the severe systemic complications observed in untreated animals, significantly promoting survival.

We first confirmed the multilineage differentiation potential of MSCs and verified the expression of characteristic surface markers by flow cytometry, together with normal genomic stability by karyotype analysis. Subsequently, MSC-EVs were successfully isolated and exhibited the typical cup-shaped morphology under transmission electron microscopy, as well as the expected expression profile of canonical exosomal markers, consistent with previous studies from our group (Costa-Ferro et al., 2024; Costa-Ferro et al., 2025). Importantly, we demonstrated that MSC-EVs promote functional improvements when administered in the acute phase of SCI in rats.

A key mechanism underlying these functional improvements appears to be the modulation of the post-injury inflammatory environment. We observed that EV treatment robustly reduced microglia reactivity and production of pro-inflammatory cytokines such as IL-1β and TNF-α while enhancing anti-inflammatory (IL-10) and neurotrophic (BDNF) factors both in the acute and subacute phases after SCI model. In SCI, IL-10 can suppress the release of pro-inflammatory factors and reduce neuronal cell apoptosis, thereby promoting the functional recovery (Yang et al., 2016) and the reduced levels of pro-inflammatory cytokines (IL-1β and TNF-α) observed in our study agree with previous research has demonstrated that these vesicles can mitigate neuroinflammation via various mechanisms (Huang et al., 2017; Sun et al., 2018; Fan et al., 2021).

This dual effect suggests that EVs not only suppress inflammation but also promote a regenerative microenvironment that may facilitate neuronal survival and plasticity. These results are consistent with previous reports highlighting the immunomodulatory and trophic cargo of MSC-EVs, including miRNAs (Wang et al., 2023; Lai et al., 2022) and growth factors known to attenuate neuroinflammation and support axonal repair. Zhang Y. et al. (2021) found that bone marrow-derived MSC-EVs inhibited the level of inflammatory factors in SCI rats, thereby protecting the damaged neurons and promoting the recovery of motor function in rats (Zhang M. et al., 2021).

Histological analyses further revealed that EV therapy reduced lesion cavity formation, immune cell infiltration, tissue necrosis, and astrocytic reactivity. This attenuation of glial scar formation is particularly important, excessive astrocyte reactivity leads to the formation of the glial scar, characterized by dense fibrous tissue and high CSPG content (Park et al., 2022), which restricts regenerative axonal sprouting (Schiera et al., 2024) and reactive astrocytes are major contributors to secondary damage and inhibitors of axonal regeneration after SCI (Karimi-Abdolrezaee et al., 2012; Romanelli et al., 2019) demonstrated that intravenous administration of hUC-MSCs in female rats after traumatic spinal cord injury (tSCI), more effective than their parental cells, markedly attenuates astrogliosis and fibroglial scar formation after tSCI, suggesting that the therapeutic effect is primarily mediated by secreted factors rather than cellular integration.

Another important aspect of our study is the biodistribution profile of EVs. *In vivo* imaging revealed a selective accumulation of EVs at the lesion site, with peak retention observed at day 7 post-injection. Their sustained retention at the lesion could enhance therapeutic efficacy by prolonging the availability of bioactive cargo during the critical window of secondary injury progression. However, previous studies have shown that systemically administered EVs can also modulate cytokine release and promote neuroprotection and functional recovery following SCI (Huang et al., 2017).

Taken together, our results support the concept that EV therapy acts through a multifaceted mechanism: (i) modulating systemic and local inflammation, (ii) enhancing neurotrophic support, (iii) preserving tissue integrity, and (iv) sustaining the delivery of bioactive molecules at the injury site. These combined effects contribute to improved functional outcomes after SCI. Importantly, the survival benefit observed in EV-treated animals highlights their translational relevance, as systemic complications are a major determinant of mortality in SCI patients; traumatic spinal cord injuries have reduced overall life spans and higher mortality, especially in the first year after an injury, compared with the general population. Patients with traumatic spinal cord injuries have reduced overall life spans and higher mortality, especially in the first year after an injury, compared with the general population (Ahuja et al., 2017).

Although survival in rodent models cannot be directly extrapolated to human clinical outcomes due to species-specific differences in physiology, systemic responses, and post-injury care, its inclusion as an endpoint provides complementary translational value. Most preclinical studies of SCI report only functional or histological improvements, whereas survival integrates systemic factors such as neurogenic shock, systemic inflammation, and acute respiratory dysfunction (Zhang Y. et al., 2021). These processes are also associated with early mortality in patients (Shao et al., 2011). Therefore, the improved survival observed in EV-treated animals likely reflects a broader modulation of the acute systemic response to injury rather than a mere local effect at the lesion site, reinforcing the overall robustness of the therapeutic benefit.

This study has some limitations that should be acknowledged. First, we evaluated the overall therapeutic effects of hUCMSC-derived EVs in SCI but did not dissect the contribution of specific EV cargo (e.g., miRNAs, proteins, lipids) to the observed outcomes. Such mechanistic studies will be essential to better understand and refine their therapeutic potential. Second, we used a single dose and route of administration, which precludes conclusions about optimal dosing strategies or alternative delivery methods. Third, EVs were administered only in the acute phase after SCI, and their potential efficacy in subacute or chronic stages of injury remains to be determined.

## 5 Conclusion

In conclusion, this study demonstrates that hUCMSC-derived EV therapy exerts neuroprotective effects after SCI, characterized by reduced inflammation, attenuated glial scar formation, and improved functional recovery. Importantly, EV administration was also associated with enhanced survival, a clinically relevant outcome that has been rarely addressed in preclinical SCI studies. Together, these findings provide a compelling rationale for further investigation of EV-based approaches, including mechanistic studies, optimized dosing strategies, and evaluation across different stages of injury, to advance their development as a promising therapeutic strategy for spinal cord injury.

## Data Availability

The raw data supporting the conclusions of this article will be made available by the authors upon reasonable request.
